# Assessment of the dynamic range in dynamic contrast-enhanced magnetic resonance imaging breast examinations

**DOI:** 10.1186/bcr3294

**Published:** 2012-11-09

**Authors:** AEW Ledger, M Borri, M Schmidt, R Pope, E Scurr, T Wallace, C Richardson, M Usher, S Allen, R Wilson, K Thomas, N deSouza, MO Leach

**Affiliations:** 1 CR-UK and EPSRC Cancer Imaging Centre, Institute of Cancer Research and Royal Marsden NHS Foundation Trust, Sutton, UK; 2 Department of Radiology, Royal Marsden Hospital, Sutton, UK; 3 Clinical Research and Development, Royal Marsden Hospital, Sutton, UK

## Introduction

Accurate dynamic contrast-enhanced magnetic resonance imaging (DCE-MRI) protocol evaluation is necessary to ensure reliable classification of contrast-agent (CA) uptake curves. This work presents a novel retrospective method to assess the dynamic range that constructs CA uptake curves using enhancement of the internal mammary artery.

## Methods

Routine clinical breast examinations were performed using 3D fat-suppressed spoiled gradient-echo sequences (1.5T). Retrospective analysis was approved by the Clinical Audit Committee. Five different protocols were evaluated (10°, 14° and 18° flip angles (FAs), radial or linear k-space sampling), with seven to 10 patients in each group (*n *= 45). CA uptake curves were constructed from a standardised axial slice through the right mammary artery, and maximum relative enhancement (*E_max_*) and time-to-peak enhancement (*T_max_*) were measured for each examination [1]. *E_max _*and *T_max _*were compared between protocols (ANOVA/Mann-Whitney; *P *< 0.05 indicating significance). For each protocol, calculated values of maximum relative enhancement (*E_calc_*) were derived from the Bloch equations and compared with *E_max _*to validate the results.

## Results

A lower FA and radial k-space sampling resulted in a statistically significant decrease in *E_max _*(*P *< 0.0001 and *P *= 0.001, respectively). Radial protocols exhibited greater *T_max _*than linear protocols at FAs of both 14° (*P *= 0.025) and 18° (*P *< 0.0001). *E_calc _*was found to increase with FA as expected, with good agreement between *E_calc _*and *E_max_*.

## Conclusion

Significant differences were found between patient groups with only small alterations in protocol. Observations agreed with expected results, validating this method of retrospective analysis. The dynamic range is optimised at higher flip angles and with linear k-space sampling.
